# Severity patterns in chronic otomastoiditis: a retrospective single-center study

**DOI:** 10.25122/jml-2026-0145

**Published:** 2026-09

**Authors:** Alexandra Mădălina Bizdu-Branovici, Luana Gherasie, Maria Denisa Zica, Andreea Rusescu, Irina Gabriela Ioniță, Razvan Hainaroșie, Viorel Zainea

**Affiliations:** 1 General Medicine, Carol Davila University of Medicine and Pharmacy, Bucharest, Romania; 2 Prof. Dr. D. Hociotă Institute of Phonoaudiology and Functional ENT Surgery, Bucharest, Romania

**Keywords:** chronic otomastoiditis, chronic otitis media, complications, computed tomography, magnetic resonance imaging, vertigo, CT, computed tomography, IQR, interquartile range, MRI, magnetic resonance imaging, SD, standard deviation, STROBE, Strengthening the Reporting of Observational Studies in Epidemiology

## Abstract

Chronic otomastoiditis can cause local structural destruction and clinically important complications. This retrospective single-center study described severity patterns and diagnostic investigation use during 2020–2024. The analysis included 137 clinical records of chronic otomastoiditis. Clinical complications were distinguished from structural defects and ossicular erosion. Frequencies, descriptive between-group differences, and missing-data sensitivity analyses were calculated. Inferential tests were not applied because patient-level independence could not be verified throughout the dataset. Mean ± standard deviation age was 41.8 ± 16.4 years. At least one clinical complication was present in 13/136 (9.6%); an expanded endpoint including tegmen dehiscence and posterior external auditory canal wall fistula was positive in 15/136 (11.0%). Facial paralysis occurred in seven records and semicircular canal fistula in five. Computed tomography was performed in 131/137 (95.6%), and magnetic resonance imaging in 17/137 (12.4%). Complications occurred in 8/17 (47.1%) records with vertigo versus 5/119 (4.2%) records without vertigo. Corresponding proportions with and without magnetic resonance imaging were 4/17 (23.5%) and 7/71 (9.9%). Microbiological information was interpretable in 39 records, with organisms identified in 25. Structural injury and clinical complications represented different dimensions of severity. Vertigo showed a marked descriptive association with complications. Imaging comparisons were limited by indication bias and missing information and did not establish diagnostic accuracy or independent predictors.

## INTRODUCTION

Chronic middle-ear and mastoid disease ranges from persistent local inflammation to destructive lesions and extracranial or intracranial complications. Different manifestations of severity are not interchangeable: ossicular erosion, tegmen defects, facial paralysis, and intracranial infection have different implications for assessment and management. Computed tomography (CT) and magnetic resonance imaging (MRI) address complementary anatomical and tissue-characterization questions [1].

Recent surgical studies have examined labyrinthine fistula, dural exposure, and facial canal dehiscence as distinct findings rather than a single homogeneous outcome [2]. This distinction is also important when interpreting routine clinical datasets. A high frequency of imaging does not itself demonstrate detection of a lesion, and a structural defect does not necessarily indicate infectious extension.

This study aimed to describe clinical complications and structural injury in chronic otomastoiditis and examine their descriptive relationships with symptoms and diagnostic investigation use.

## MATERIAL AND METHODS

### Design and study records

This retrospective single-center observational study covered January 1, 2020, to December 31, 2024, at the Prof. Dr. D. Hociotă Institute of Phonoaudiology and Functional ENT Surgery, Bucharest, Romania. Reporting follows the relevant principles of the Strengthening the Reporting of Observational Studies in Epidemiology (STROBE) statement [3]. Chronic otomastoiditis denotes the chronic middle-ear and mastoid disease category used in the clinical dataset; the analysis does not assume that every record represents independently adjudicated cholesteatoma.

Clinical selection considered the relevance of otic disease, the operative context, and sufficient essential information to characterize the episode. Admissions for unrelated disease, routine review, or exclusively medical treatment were outside the intended surgical selection. Missing CT or MRI information alone was not an exclusion criterion. Same-day episodes required distinction between routine day attendance and an emergency operation followed by transfer.

The selected clinical table contained 191 records. Eight confirmed duplicate copies were removed, and four records were excluded because the assessed episode involved conservative treatment or evaluation without a confirmed otic operation. One additional eligible acute emergency episode, involving urgent otologic surgery followed by same-day neurosurgical transfer, was included from the clinical source documentation. The source dataset comprised 180 clinical records: 43 acute and 137 chronic. This article analyzes only the 137 records of chronic otomastoiditis; the 43 acute records were not included in any of its analyses. This selection flow starts with the selected clinical table, not an unverified total of raw registry exports.

The unit of analysis was the retained clinical record. Revisions, reoperations, and contralateral operations may represent separate episodes. The available linkage identifiers did not establish all person-level relationships reliably. Records were therefore not treated as proven independent patients, and no first-admission rule or inferred patient grouping was introduced. Author-validated clinical entries without a secure photographic match were retained. Operative text was preserved without interpreting a past operation as proof of surgery during the current episode.

### Variables and outcomes

Variables included age, sex, year, laterality, length of stay, symptoms, otological examinations, CT, MRI, ossicular erosion, individual complications, biological measurements, and treatment information. Complication codes were clinically checked by the investigator: 1 indicated presence and 0 absence. Blank cells remained unavailable. For investigations, 1 and 0 indicated performed and not performed, respectively; these codes did not represent imaging-positive and imaging-negative results. Free-text diagnoses were not converted automatically by keyword matching.

During clinical verification, an explicitly affirmed complication in the free-text diagnosis was coded as present. Ambiguous terms, differential diagnoses, and unrelated historical findings were not treated as current complications. Microbiological information was grouped as an identified organism, a negative result, nonspecific or nonpathogenic flora, or unavailable/uninterpretable information. Multiple organisms could be reported for one record. A missing culture result did not indicate a negative culture.

The primary endpoint was at least one clinical complication among facial paralysis, semicircular canal fistula, retroauricular abscess, cervical abscess, temporozygomatic abscess, cerebral abscess, meningitis, venous sinus thrombosis, epidural abscess, subdural abscess, subdural empyema, and hydrocephalus. The composite was positive if any component was positive, negative only if every component was negative, and otherwise indeterminate. Each record contributed at most once to the composite.

Tegmen dehiscence and posterior external auditory canal wall fistula were reported separately and added to a secondary expanded endpoint of clinical complications or structural lesions. Ossicular erosion was not included in either endpoint. Subdural abscess and empyema labels were retained from the source, but overlapping labels did not produce duplicate counts in the composite. Outcomes were not interpreted as postoperative adverse-event rates.

### Statistical analysis

Categorical variables are reported as counts and percentages with explicit denominators. Continuous variables are summarized by mean and standard deviation (SD), median and interquartile range (IQR), and range. Quartiles use linear interpolation. Available-case comparisons report absolute differences in proportions and descriptive prevalence ratios, without implying prospective risk or adjustment for confounders [4]. Calculations used Python 3.12 with pandas and NumPy; an independent standard-library implementation checked counts and summaries directly against the Excel file structure.

No inferential significance tests, confidence intervals based on independent observations, or multivariable models were fitted because patient-level independence and clustering could not be verified. Statistical significance was therefore not assessed. Clinical interpretation considers the magnitude of differences, subgroup size, and missing information; it is not inferred from a *P* value threshold [5].

Missing values were not imputed. For the single record with unknown composite status, sensitivity analyses considered both possible outcomes. For MRI, all possible allocations of missing investigation status among records with known outcomes were enumerated. These produce deterministic bounds, not confidence intervals. C-reactive protein values below a reporting limit were not converted into exact numbers.

## RESULTS

### Characteristics of the chronic series

The analysis included 137 chronic records. Mean ± SD age was 41.8 ± 16.4 years, with a median of 39.0 years (IQR, 29.0–55.0) and a range of 7–81 years. Records of male and female patients accounted for 74/137 (54.0%) and 63/137 (46.0%), respectively. Length of stay was available for 133 records, with a median of 11.0 days (IQR, 9.0–13.0; Table 1).

**Table 1:** Characteristics and presenting symptoms

Characteristic	Chronic records (*n* = 137)
Age (years), mean ± SD	41.8 ± 16.4
Age (years), median (IQR)	39.0 (29.0–55.0)
Male/female, *n*	74 / 63
Right / left / bilateral involvement, *n*	86 / 41 / 8
Laterality unavailable, *n*	2
Length of stay (days), median (IQR)	11.0 (9.0–13.0)
**Presenting symptoms, *n/N* (%)**	
Hearing loss	137/137 (100.0%)
Otorrhea	127/137 (92.7%)
Vertigo	17/137 (12.4%)
Tinnitus	19/134 (14.2%)
Otorrhagia	3/137 (2.2%)

Length of stay was available in 133 records. Symptom proportions use the stated available denominator. IQR, interquartile range; *n*, number of records with the characteristic; *N*, number of records with available information; SD, standard deviation.

### Clinical complications and structural lesions

The clinical-complication endpoint was positive in 13/136 (9.6%), negative in 123 records, and indeterminate in one. The expanded endpoint was positive in 15/136 (11.0%). Facial paralysis and semicircular canal fistula were the most frequent individual clinical complications (Table 2). No intracranial complication was positive in the chronic subset; one record had an indeterminate intracranial composite because hydrocephalus status was missing. Zero observed events do not imply zero clinical risk.

**Table 2:** Complications and structural findings

Finding	Present	Missing, *n*
**Clinical complications**		
Facial paralysis	7/137 (5.1%)	0
Semicircular canal fistula	5/137 (3.6%)	0
Retroauricular abscess	1/137 (0.7%)	0
Temporozygomatic abscess	1/137 (0.7%)	0
**Structural findings**		
Tegmen dehiscence	3/137 (2.2%)	0
Posterior external auditory canal wall fistula	4/137 (2.9%)	0
Ossicular erosion	72/116 (62.1%)	21
**Composite endpoints**		
Any clinical complication	13/136 (9.6%)	1
Expanded endpoint	15/136 (11.0%)	1

Findings can coexist. Expanded endpoint: clinical complications, tegmen dehiscence, or posterior external auditory canal wall fistula. Ossicular erosion is reported separately and is not included in either endpoint.

Ossicular erosion was present in 72/116 (62.1%) and unavailable in 21 records. Its higher frequency than the clinical-complication endpoint illustrates that local structural injury and clinical extension are different dimensions of severity. Figure 1 displays the nonzero clinical complications without adding structural findings to their count.

**Figure 1:**
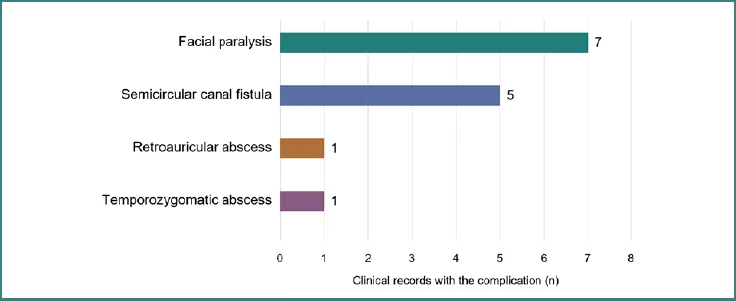
Nonzero clinical-complication categories in 137 chronic records. Categories may coexist and should not be summed to obtain the number of records with any complication. Structural defects and ossicular erosion are excluded from this figure. n, number of records.

### Diagnostic investigation use

Otoscopy was performed in 136/137 (99.3%), microscopic examination in 121/137 (88.3%), and endoscopic examination in 12/137 (8.8%). CT was performed in 131/137 (95.6%) and MRI in 17/137 (12.4%). CT status was unavailable in four records and MRI status in 48. Every MRI record also indicated CT, although the order of examinations was not established.

Among records with both variables known, complications occurred in 11/130 (8.5%) records with CT and 0/2 (0.0%) records without CT. The group without CT contained only two records. With MRI, the proportion was 4/17 (23.5%), compared with 7/71 (9.9%) without MRI, a descriptive difference of 13.7 percentage points (Table 3).

**Table 3:** Exploratory comparisons for clinical complications

Feature	Present or performed	Absent or not performed	Difference (percentage points)
CT	11/130 (8.5%)	0/2 (0.0%)	8.5
MRI	4/17 (23.5%)	7/71 (9.9%)	13.7
Vertigo	8/17 (47.1%)	5/119 (4.2%)	42.9
Otorrhea	12/126 (9.5%)	1/10 (10.0%)	−0.5
Tinnitus	3/19 (15.8%)	8/114 (7.0%)	8.8
Ossicular erosion	6/71 (8.5%)	0/44 (0.0%)	8.5

The numerator is the number of records with a clinical complication; denominators require both variables to be known. Differences are descriptive and unadjusted; no statistical tests were applied, and statistical significance was not assessed. CT, computed tomography; MRI, magnetic resonance imaging.

### Symptoms and severity comparisons

Vertigo showed a descriptive association with complications, which were present in 8/17 (47.1%) records with vertigo versus 5/119 (4.2%) records without vertigo. The difference was 42.9 percentage points and the descriptive prevalence ratio 11.20. Hearing loss was universal and therefore had no symptom-negative comparison group.

Age and hospital stay were also described by complication status. Median age was 43.0 years in the complication group and 37.0 years in the group without complications. Median stay was 11.0 versus 10.0 days, based on 12 and 120 evaluable records, respectively. These differences do not define diagnostic thresholds or quantify the total burden of care across other admissions.

### Microbiological findings

Microbiological information was interpretable in 39 of 137 records (28.5%). Organisms were identified in 25 of these 39 records (64.1%), results were negative in 10 (25.6%), and nonspecific or nonpathogenic flora was reported in four (10.3%). Information was unavailable in 96 records; two additional entries contained a numeric code without an identifiable organism or interpretable result. Table 4 describes the available findings without extending their distribution to the whole chronic series.

**Table 4:** Microbiological findings in records with interpretable information

Result or organism category	Records, *n/N* (%)
**Result category**	
Identified organism	25/39 (64.1%)
Negative result	10/39 (25.6%)
Nonspecific or nonpathogenic flora	4/39 (10.3%)
**Organism category**	
*Staphylococcus aureus*	10/39 (25.6%)
*Proteus* spp.	6/39 (15.4%)
*Candida* spp. or other yeasts	7/39 (17.9%)
*Pseudomonas* spp.	2/39 (5.1%)
*Escherichia coli*	2/39 (5.1%)
White staphylococci or coagulase-negative staphylococci	5/39 (12.8%)

All percentages use the 39 records with interpretable microbiological information. The three result categories are mutually exclusive; organism categories can coexist. White staphylococci retain the nonspecific source terminology and were not assigned to a species. No antimicrobial susceptibility distribution was available. Values are descriptive; no statistical tests were applied.

These findings describe the microorganisms recorded during diagnostic assessment. Because sampling sites, collection timing, preceding antimicrobial treatment, and susceptibility results were not consistently available, the data do not establish causal pathogens or an empirical antibiotic recommendation. Yeast detection alone was not used to diagnose invasive fungal disease.

### Sensitivity analyses

If the single indeterminate clinical composite were classified as negative or positive, the complication proportion would be 13/137 (9.5%) or 14/137 (10.2%), respectively. The primary estimate remains 13/136 (9.6%), and the record remains in the dataset. These scenarios do not alter the source values.

For MRI status, two complicated and 46 noncomplicated records had missing information. Across all possible allocations, the difference in complication proportions between MRI-performed and MRI-not-performed groups ranged from −6.0 to 25.6 percentage points. These deterministic extremes are not equally likely assumptions, but show that the direction of the descriptive imaging association is not secure.

## DISCUSSION

This study describes a selected chronic otomastoiditis series with frequent otorrhea, universal hearing loss, substantial ossicular injury, and a smaller subgroup with clinical complications. The separation of structural changes from clinical extension is important: a destructive local lesion can affect operative planning without belonging to the same outcome category as an abscess or neurological complication.

The semicircular canal fistula proportion of 5/137 (3.6%) is broadly within the range reported in recent surgical literature, although the populations and units of analysis differ. Chang *et al*. analyzed 189 patients with 191 operated ears and reported labyrinthine fistula in 7.4% of cholesteatoma cases and 2.4% of mastoiditis cases [2]. Our mixed chronic series was not stratified by independently adjudicated cholesteatoma, and a record-level proportion cannot be treated as an equivalent ear-level rate. Facial canal dehiscence in that study should not be equated with facial paralysis in ours.

Atchariyasathian and Tangchitnob reported 55 patients with complications among 4364 patients with chronic otitis media over 20 years, an overall proportion of 1.26% [6]. The 9.6% proportion in our available-case analysis is not evidence of a higher institutional population risk. Our denominator is a selected clinical series, definitions are not identical, and repeat-episode structure is not fully resolved. The comparison supports the clinical relevance of complications but does not establish equal rates or a shared numerical trend.

The pattern of extensive CT use and selective MRI use is compatible with their complementary diagnostic roles [1]. However, our data identify whether a method was performed, not which structures it correctly characterized. The high CT frequency and the very small non-CT group prevent meaningful conclusions about comparative benefit. The higher complication proportion in the MRI group may reflect indication bias, while missing MRI status can reverse the descriptive difference.

The organisms recorded in our series included *Staphylococcus aureus, Proteus* spp., and *Pseudomonas* spp., also reported in the 2025 complication series [6]. Our microbiological subset additionally contained *Candida* or other yeasts. Differences in sampling and case selection prevent comparison of organism frequencies or susceptibility patterns. These findings provide a microbiological description of the available records rather than evidence of an association between a specific organism and complications.

Vertigo showed the largest descriptive difference among the symptoms examined. This observation is clinically coherent with vestibular involvement, but should not be translated into a diagnostic rule. The composite itself includes semicircular canal fistula, and symptom–lesion overlap may contribute to the association. There was no standardized prospective symptom assessment, lesion-level test reference, or external validation.

The absence of a positive intracranial event in this chronic subset differs from reports that include such complications [6]. It does not refute those reports and does not establish that intracranial extension is absent from the institute’s practice. The acute case of sigmoid sinus thrombosis with cerebellar abscess and hydrocephalus is included once in the full doctoral cohort, but not in this chronic-disease subset. All acute records remain outside the article’s scope.

### Limitations

The retrospective, single-center design and clinical selection limit generalizability. Records were clinically validated, but the analytical file did not support complete person-level linkage. Removing duplicate copies does not establish independence of all retained episodes; therefore, conventional significance tests and independent-observation confidence intervals were not reported. This is a descriptive and hypothesis-generating study, not a confirmatory risk-factor analysis.

Imaging results, protocols, and timing were not uniformly available. There was no common reference standard for all lesions, so sensitivity, specificity, predictive values, and imaging–operative concordance could not be estimated. Missing investigation and biomarker information may be associated with disease severity or documentation. The surgical context should not be interpreted as independently proven current-episode surgery in every retained record. These constraints delimit the conclusions without converting missing data into negative findings.

## CONCLUSION

In 137 chronic otomastoiditis records, clinical complications were present in 13/136 (9.6%), whereas ossicular erosion and structural defects represented separate aspects of severity. CT predominated in diagnostic assessment, and MRI was used in a smaller subgroup. Vertigo showed a marked descriptive association with complications, but imaging and symptom comparisons do not establish independent predictors or diagnostic accuracy. The findings support careful integration of clinical assessment and imaging within the limits of this selected retrospective series.

## Data Availability

The record-level dataset contains clinical information and is not publicly available. Any access is subject to institutional authorization and applicable confidentiality requirements.
